# Characterization of Methicillin-Resistant and -Susceptible Staphylococcal Isolates from Bovine Milk in Northwestern China

**DOI:** 10.1371/journal.pone.0116699

**Published:** 2015-03-10

**Authors:** Longping Li, Luoxiong Zhou, Lihong Wang, Huping Xue, Xin Zhao

**Affiliations:** 1 College of Animal Science and Technology, Northwest A&F University, Yangling, Shaanxi, People’s Republic of China; 2 Department of Animal Science, McGill University, 21,111 Lakeshore, Ste. Anne de Bellevue, Quebec, H9X 3V9, Canada; Rockefeller University, UNITED STATES

## Abstract

Emergence of methicillin-resistant *Staphylococcus aureus* (MRSA) and methicillin-resistant coagulase-negative staphylococci (MR-CoNS) in bovine milk is a major public health concern. The primary purpose of this research was to determine molecular genetic characteristics and antibiotic resistance of staphylococcal isolates recovered from milk of mastitic cows in the Shaanxi Province in Northwestern China. One hundred and thirteen methicillin-susceptible *Staphylococcus aureus* (MSSA), one *mecA*-positive and phenotype-positive MRSA, seven *mecA*- and *mecC*- negative but phenotype-positive MRSA and two MR-CoNS including one oxacillin-susceptible *mecA*-positive *Staphylococcus haemolyticus* (OS-MRSH) and one *mecA*-positive and methicillin-resistant *Staphylococcus epidermidis* (MRSE) isolates were recovered from 214 quarter milk samples on 4 dairy farms. All above 123 isolates were subjected to antibiotic resistance profiling. *S*. *aureus* isolates were also genotyped using the *spa* typing and the multilocus sequence typing (MLST). Eight MRSA and 2 MR-CoNS isolates were additionally tested for SCC*mec* types. Resistance was common among isolates against ampicillin or penicillin (80.5%), kanamycin (68.3%), gentamicin (67.5%), tetracycline (43.9%) and chloramphenicol (30.1%). However, no isolate was resistant to vancomycin or teicoplanin. Twenty, 29 and 58 isolates showed resistance to 1, 2 or more than 2 antibiotics, respectively. The predominant multidrug resistance profile was penicillin/ampicillin/kanamycin/gentamicin/tetracycline (46 isolates). Most *S*. *aureus* isolates belonged to *spa* types t524 (n = 63), t11772 (a new type, n = 31) and t4207 (n = 15). At the same time, MLST types ST71 (n = 67) and ST2738 (a new type, n = 45) were identified as dominant sequence types. The *mecA*-positive and phenotype-positive MRSA isolate had a composite genotype t524-ST71-SCC*mec*IVa, while 7 *mecA*-negative but phenotype-positive MRSA isolates were all t524-ST71. The OS-MRSH isolate contained a type V SCC*mec* cassette, while the MRSE isolate possessed a non-typeable SCC*mec*. The *spa*-MLST types t11772-ST2738 (n = 27), t11807-ST2683 (n = 4) and t11771-ST2738 (n = 3) were newly identified genotypes of *S*. *aureus*. These new genotypes and multidrug-resistant staphylococci could pose additional threat to animal and human health.

## Introduction

Milk and milk products are common vehicles of staphylococcal food poisoning [1]. Appearance of methicillin-resistant *S*. *aureus* (MRSA) in milk and dairy foods has become a major concern for animal and human health. While both MRSA and MSSA can acquire many antimicrobial determinants, MRSA are resistant to practically all β-lactam antibiotics [2]. In addition, the livestock-associated (LA) MRSA with the ST398 genotype has been detected among swine farmers [3]. Genetic analyses of MRSA isolated from human and bovine mastitis have suggested that horizontal gene transfer between human and animal pathogens can occur [4]. Previous studies have reported appearance of MRSA in bovine mastitic milk in several countries, including Switzerland [5], Korea [6], Belgium [7] and Turkey [8]. There have been a few reports on MRSA from bovine milk in China. Recently, a high prevalence of MRSA (47.6%) was reported from dairy cows with clinical mastitis in China [9], while another study reported no MRSA isolates in raw milk samples [10]. In addition to MRSA, methicillin-resistant coagulase-negative staphylococci (MR-CoNS) from mastitic milk have been reported in various countries [11, 12]. However, there is no report in the literature about MR-CoNS from milk in China.

The main objective of this research, therefore, was to study molecular genetic characteristics and antibiotic resistance patterns of methicillin-susceptible *Staphylococcus aureus* (MSSA), MRSA and MR-CoNS from mastitic milk samples collected from the Shaanxi Province in Northwestern China.

## Materials and Methods

### 2.1 Ethics

Milk samples were obtained from dairy cows with naturally occurring clinical mastitis under the ethical approval granted by the College of Animal Science and Technology, the Northwest Agriculture and Forestry (A&F) University (Permit Number: NWAFU1008). No specific permissions were required for these locations/activities. No invasive or pain-causing procedures were involved. All efforts were made to minimize animal suffering. This study did not involve endangered or protected species.

### 2.2 Isolation of MSSA, MRSA and MR-CoNS

Milk samples were taken from cows with clinical mastitis which was manifested with decreased milk production, color change of the milk and inflammation of udders. Selection of cows was consulted with professional veterinarians and sample collection was permitted by owners of the dairy farms. Milk samples were collected from 4 dairy farms in Baoji, Xi’an and Xianyang in the Shaanxi Province. Milk samples were collected after cleaning the teats, discarding a few streams of milk and scrubbing the teat ends with cotton balls moistened with 70% alcohol. In all, 214 individual quarter milk samples were obtained from 161 cows.

In order to isolate staphylococci, aliquots of individual milk samples were added to an equal volume of a double-strength enrichment broth (a trypticase soy broth supplemented with 10% NaCl and 1% sodium pyruvate) (Oxoid, Basingstoke, Hampshire, UK). After 24 h incubation at 35°C, the enrichment broth was streaked onto the Baird-Parker (Oxoid) agar containing 30% egg yolk with 1% tellurite (Oxoid) and onto the phenol red mannitol salt agar plates. Following 48 h incubation at 35°C, one to three presumptive staphylococcal colonies from each plate were transferred to trypticase soy agar plates. Yellow colored colonies from the phenol red mannitol salt agar plates were assumed to be *S*. *aureus*. Further identification of these presumptive staphylococcal colonies was first based on conventional methods including Gram stain staining, colony morphology, a catalase test and a coagulase test with rabbit plasma. *S*. *aureus* and MR-CoNS were finally confirmed by molecular methods described later. Meanwhile, aliquot samples were also streaked onto an oxacillin resistance screening agar base with the selective supplement SR0195E (Oxoid). MRSA appeared as intense and diffuse blue colonies, while MSSA were inhibited by the oxacillin in the media. All *S*. *aureus* isolates (MSSA and MRSA) and two MR-CoNS isolates were further analyzed.

### 2.3 Genetic confirmation of MSSA, MRSA and MR-CoNS isolates

DNA from staphylococci was prepared using a Column Bacterial DNAout kit (Tiandz Inc., Beijing, China). In brief, 1 mL of bacterial cultures in trypticase soy broth (Oxoid) after overnight incubation was pelleted by centrifugation at 8,000 x g for 1 min. The cell pellet was resuspended in 600 μL of lysostaphin (20 μg/mL) (Tiandz Inc.) and the mixture was then incubated at 37°C for 1.5 h. Total DNA was extracted according to the manufacturer’s protocol. In addition, all *S*. *aureus* isolates were confirmed by using a polymerase chain reaction (PCR) assay targeting the species-specific 16S rRNA gene and *S*. *aureus*-specific region of the thermonuclease gene (*nuc*) as described previously [13, 14]. Amplicons of 16S rRNA and *nuc* genes were sequenced and confirmation of the species was carried out if sequences showed 98% to 100% similarity using the Basic Local Alignment Search Tool (BLAST) (http://blast.ncbi.nlm.nih.gov). MR-CoNS were confirmed by sequencing and analyses of *sodA* or *gap* genes [15, 16].

The *mecA* gene, encoding the modified penicillin binding protein PBP2a, was amplified by PCR to confirm methicillin-resistant *S*. *aureus* and CoNS isolates. The *mecA*-PCR test was performed using primers designed in this study according to the reference sequence of the MRSA252 (NCBI accession number: NC_002952.2) targeting position 44947–44969 for the forward primer (5′-CCGTTCTCATATAGCTCATCATA-3′) and position 46891–46870 for the reverse primer (5′-TAGTTGTAGTTGTCGGGTTTGG-3′). The PCR amplicons of the *mecA* gene were purified from agarose gels and ligated with the pMD18-T vector. The ligation mixture was transformed into *E*. *coli* DH5a and recombinants were selected on Luria-Bertani agar containing ampicillin (100μg/mL). Recombinant plasmid DNA was purified by standard methods and subjected to sequencing and further analyses. The sequence similarity was compared using the online BLAST database (http://blast.ncbi.nlm.nih.gov). An additional PCR for *mecC* was performed [17] when the *mecA*-PCR test was negative. In addition, detection of PBP2a protein was performed using a latex agglutination test kit (Colloidal Gold) (Beijing Qing Yuan Sheng Kang Pharmaceutical Technology Co., Ltd., Beijing, China) for MRSA and MR-CoNS isolates as recommended by the manufacturer.

### 2.4 Antimicrobial susceptibility testing (AST)

All isolates were subjected to an antimicrobial susceptibility testing for 10 antibiotics by a disk diffusion method. Isolates which were susceptible, intermediate and resistant to antibiotics were discriminated in accordance with the Clinical and Laboratory Standards Institute (CLSI) criteria [18]. The plates were incubated at 35°C with an initial reading after incubation of 24 h, and a second reading after incubation of 48 h. Moreover, susceptibility tests against vancomycin were performed by using an agar dilution method according to CLSI guidelines [19]. The standard reference strains *S*. *aureus* ATCC29213 and ATCC25923 served as quality control strains in every test run. Antimicrobial susceptibility tests were performed in triplicate. The multidrug resistance was reported when a single isolate was resistant (intermediate or complete) to 3 or more unique antimicrobial drug classes.

### 2.5 Minimum inhibitory concentrations (MICs) of MRSA and MR-CoNS isolates

The MICs of oxacillin were determined using an E-test (bioMérieux, Lyon, France) or using an agar dilution method recommended by the CLSI [19]. The test was performed on all methicillin-resistant (MR) isolates. *S*. *aureus* ATCC 29213 was included as a quality control strain in each run. The MIC tests were performed in triplicate.

### 2.6 Genotyping of MSSA, MRSA and MR-CoNS isolates

All 121 *S*. *aureus* isolates were subjected to the multilocus sequence typing (MLST) and the *spa* typing. Specifically, the MLST analysis was conducted by sequencing fragments of seven housekeeping genes (*arcC*, *aroE*, *glpF*, *gmk*, *pta*, *tpi* and *yqiL*). Allele number and sequence types (STs) were assigned by using the *S*. *aureus* MLST website (http://saureus.mlst.net). The polymorphic X-region of the protein A gene (*spa*) was amplified and sequenced for the *spa* typing. The *spa* types were assigned using an online *spa* database (http://www.spaserver.ridom.de/). In addition, MRSA and 2 MR-CoNS isolates were further tested for Staphylococcal Chromosome Cassette *mec* (SCC*mec*) types as in a previous study [20].

## Results

### 3.1 Isolation of methicillin-resistant staphylococci

One hundred and twenty one *S*. *aureu*s and 336 CoNS including 2 MR-CoNS isolates were recovered from 214 milk samples. Based on the PCR results of *mec* genes, one *S*. *aureus* isolate was identified as MRSA and two CoNS isolates were identified as MR-CoNS (one was *S*. *haemolyticus* and the other one was *S*. *epidermidis*). According to the oxacillin disk diffusion test, the *S*. *haemolyticus* isolate was sensitive to this antibiotic despite being *mecA*-positive by PCR and thus this isolate was an oxacillin-susceptible *mecA*-positive *S*. *haemolyticus* (OS-MRSH). Besides, seven *S*. *aureus* isolates showed high MICs (> 4 μg/mL) to oxacillin but did not carry the *mecA* or *mecC* gene.

### 3.2 Antimicrobial susceptibility testing

Regarding the susceptibility to the antimicrobial agents tested, 80.5% of isolates showed resistance to ampicillin or penicillin, followed by 68.3% to kanamycin, 67.5% to gentamicin, 43.9% to tetracycline, 30.1% to chloramphenicol and 8.1% to oxacillin or cefoxitin. All isolates were susceptible to vancomycin and teicoplanin. One hundred and seven isolates (87%) were resistant to at least one antimicrobial, 58 (47.2%) to three or more antimicrobials and 16 isolates were susceptible to all tested antimicrobials. According to the antibiogram, the predominant multidrug resistance profile was penicillin/ampicillin/kanamycin/gentamicin/tetracycline (46 isolates).

All oxacillin and cefoxitin resistant isolates were resistant to three or more antimicrobials while remaining susceptible to vancomycin and teicoplanin. The oxacillin MIC of the OS-MRSH isolate was 1μg/mL, while all other (methicillin-resistant) MR isolates had oxacillin MICs in the range of 4–128 μg/mL.

### 3.3 Molecular characterization of MSSA and MR isolates

Most studied *S*. *aureus* isolates carried the *spa* type t524 (n = 63) followed by t11772 (n = 31) and t4207 (n = 15). Other detected *spa* types were t521 (n = 5), t11771 (n = 3) and t11807 (n = 4). Furthermore, three *spa* types, t11771, t11772 and t11807, were newly found (Table 1). The *S*. *aureus* MLST analysis revealed 4 STs: ST71, ST2683, ST97 and ST2738. ST71 (n = 67) and ST2738 (n = 45) were most common (Table 1). ST2683 (n = 4) and ST2738 (n = 45) were novel MLST types. The *S*. *aureus* isolates had 12 different *spa*-MLST type combinations (Table 2). The most frequent combinations were t524-ST71 (n = 60), t11772-ST2738 (n = 27) and t4207-ST2738 (n = 12).

**Table 1:** *spa* and MLST types and allelic profiles of *S*. *aureus* isolates.

*Spa* typing data			MLST tying data								
*Spa* type	Repeat succession	Number of isolates	ST type	Allelic profiles							Number of isolates
				*arcC*	*aroE*	*glpF*	*gmk*	*pta*	*tpi*	*yqiL*	
t524	r04-r17	63	ST71	18	1	1	1	1	5	3	67
t11772*	r07-r23-r21-r17-r34-r33-r16-r16-r16	31	ST2683*	52	386^†^	54	18	56	32	65	4
t4207	r07-r23-r12-r21-r17-r34-r33-r16-r16-r16	15	ST97	3	1	1	1	1	5	3	5
t521	r07-r23-r12-r21-r17-r34-r34-r34-r34-r33-r34	5	ST2738*	1	395^†^	1	105	11	4	10	45
t11771*	r07-r23-r21-r17-r34-r33-r16-r16	3									
t11807*	r04-r20-r17-r17-r17	4									

*Novel *spa* types or ST types.

^†^Novel alleles.

**Table 2:** Numbers of *S*. *aureus* isolates with different combinations of *spa* and MLST types.

*spa*-MLST type	Number of isolates
t11772-ST2738	27
t4207-ST2738	12
t11771-ST2738	3
t524-ST71	60
t11772-ST97	1
t4207-ST71	3
t524-ST2738	2
t11772-ST71	3
t521-ST97	3
t524-ST97	1
t11807-ST2683	4
t521-ST71	2

The *mecA*-positive MRSA, JY39–1, showed a composite profile of t524-ST71-SCC*mec*IVa. Seven *mecA*-negative but phenotype-positive MRSA isolates also shared the t524-ST71 profile. The OS-MRSH isolate contained a SCC*mec* V type element, while the SCC*mec* element of the MRSE isolate was untypable by the method used in this study.

## Discussion

Investigation of MRSA from farm animals has been intensified all over the world in recent years. However, limited information is available for genetic characterization of MRSA and MR-CoNS from dairy cows in China. In the present study, using phenotypic and genotypic methods, 113 MSSA, 1 *mecA* positive MRSA (JZY39–1), 1 OS-MRSH (NW19), 1 MRSE (JZ3–2), 7 *mecA*-negative but phenotype-positive MRSA and 336 MS-CoNS were isolated from 214 milk samples.

Surprisingly, 7 phenotype-positive MRSA isolates in this study did not carry the *mecA* or *mecC* gene. The MecA-negative but phenotype-positive MRSA have been previously reported. For example, 15 out of 18 and 3 out of 13 phenotype-positive MRSA isolates did not carry the *mecA* gene in Turkey [21] and India [22], respectively. Recently, the presence of *mecA*-negative MRSA strains in bovine milk samples has also been reported in China [23]. In a previous study, a number of novel amino acid substitutions have been found in the transpeptidase domains of PBPs 1, 2 and 3 in phenotype-positive MRSA isolates that lacked the *mecA* or *mecC* gene [24]. Another study reported that a *mecA*-negative strain of MRSA with high-level β-Lactam resistance contained five single-nucleotide polymorphisms in three genes, specifically, those encoding PBP4, a low-molecular-weight penicillin-binding protein, GdpP, a predicted signaling protein, and AcrB, a cation multidrug efflux transporter [25]. The possibility for existence of such mutations in our isolates will be further studied.

Two MR-CoNS, one MRSE and one OS-MRSH, were isolated in this study. MR *S*. *epidermidis* or MR *S*. *haemolyticus* have been reported from mastitic milk samples in Switzerland [11], Turkey [26] and Korea [27]. To the best of our knowledge, this is the first report of OS-MRSH of bovine origin. Recently, one study reported that 37 of the 49 *mecA*-positive isolates from bovine mastitic milk were susceptible to oxacillin as determined by antimicrobial susceptibility assays and were thus classified as oxacillin-susceptible *mecA*-positive *S*. *aureus* (OS-MRSA) [9]. Both OS-MRSA and OS-MRSH are very difficult to detect by routine phenotypic methods, because of their susceptibility to oxacillin and cefoxitin. These bacteria could turn into more resistant subpopulations when exposed to β-lactams [28].

The antimicrobial resistance level in our study was relatively high. Fifty-eight out of 123 staphylococcal isolates were resistant to 3 or more antimicrobial classes including most of the commonly used antimicrobials in the region. Several clinical studies have reported lower levels of antimicrobial resistance among mastitic *S*. *aureus* in the United States and European countries [2]. For example, 49.6% of *S*. *aureus* from bovine mastitis in the United States during 1994 to 2000 were resistant to ampicillin or penicillin, 1.1% to gentamicin and 8.5% to tetracycline [29]. The uncontrolled antibiotic use in the region could be a reason for spread of multidrug-resistant isolates.

New genotypes of bovine milk associated MSSA and MRSA clones were found for the first time in the present study. Three *spa* types, t11772, t11771 and t11807 and two STs, ST2683 and ST2738, were newly found. Moreover, the main *spa* types from this study were t524, t11772 and t4207, while the main *spa* types for bovine mastitis associated *S*. *aureus* isolates in the Netherlands and Denmark were t529, t524, t518 or t543 [30, 31]. The most frequent combination of *spa*-MLST types was t524-ST71, including 1 MRSA, 7 *mecA*-negative but phenotype-positive MRSA and 52 MSSA isolates. The t524-ST71 *S*. *aureus* from clinical and subclinical bovine mastitis were also prevalent throughout France, Netherlands, Denmark and Switzerland (http://www.spaserver.ridom.de/; http://saureus.mlst.net/) but they were all methicillin susceptible [30, 31]. How our 8 t524-ST71 isolates became resistant to methicillin is unknown at this stage and it deserves further investigation.

## Conclusion

Three new *spa* types (t11772, t11771 and t11807) and two ST types (ST2683 and ST2738) were found in this study. One OS-MRSH isolate carried the *mecA* gene but was susceptible to oxacillin. In addition, 7 *mecA*-negative but phenotype-positive *S*. *aureus* isolates shared the t524-ST71 profile. These new genotypes of isolates with a high level of antimicrobial resistance could pose additional threat to animal and human health.
